# COVID–19-associated diffuse dermal angiomatosis managed with pentoxifylline and topical timolol

**DOI:** 10.1016/j.jdcr.2023.07.002

**Published:** 2023-07-14

**Authors:** Michelle Toker, Daniel R. Antohi, Pooja Srivastava, Bijal Amin, Benedict Wu

**Affiliations:** a Division of Dermatology, Montefiore Medical Center, Albert Einstein College of Medicine, Bronx, New York; b Department of Pathology, Montefiore Medical Center, Albert Einstein College of Medicine, Bronx, New York

**Keywords:** COVID-19, dermatopathology, diffuse dermal angiomatosis, pentoxifylline, SARS-CoV-2 infection, topical timolol, vasculopathy

*To the Editor:* We appreciate the article from Young et al,^1^ which described gestational gigantomastia as a risk factor for diffuse dermal angiomatosis (DDA), allowing us to introduce another unreported risk factor and treatment options. DDA is a cutaneous reactive angiomatosis presenting as reticulated erythematous-to-violaceous plaques with ulceration. DDA of the breasts (DDA-B) typically occurs in middle-aged women with the following risk factors: obesity, large pendulous breasts, smoking, hypertension, and vasculopathy.^2^ Macromastia coupled with vaso-occlusive conditions or hypercoagulability leads to prolonged ischemia and reactive vascular proliferation.^3^ DDA-B after COVID-19 has not been reported. We present a case of DDA-B after COVID-19 infection and suggest adding topical timolol to the therapeutic armamentarium.

A 39-year-old woman, unvaccinated against COVID-19, with a high body mass index (64.01 kg/m^2^), and size “M” bra-cup presented with a 2-month history of painful rash affecting both breasts. A medical history review was negative for hypertension, smoking, miscarriages, thromboembolic disorders, and prothrombotic medications. She first noticed the skin lesions ∼1 month after COVID-19 infection, confirmed via polymerase chain reaction nasal swab. On physical examination, the distal aspect of the breasts exhibited livedoid changes with crusted erosions (Figs 1, *A* and 2, *A*). Laboratory analysis at initial visit revealed thrombocytosis with normal prothrombin time, partial thromboplastin time, and D-dimer. Her antiphospholipid and antinuclear antibodies were negative. An 8-mm punch biopsy of an erosion revealed vascular proliferation highlighted by CD34 (Fig 3) with negative human herpesvirus-8 stain. Clinicopathologic correlation confirmed DDA-B and initiated pentoxifylline 400 mg daily. There was partial improvement at 1-month follow-up, with evidence of re-epithelialization (Figs 1, *B* and 2, *B*). Topical timolol 0.5% solution applied twice daily to the affected areas was added. After 6 weeks of pentoxifylline and timolol, the treated areas completely re-epithelialized (Figs 1, *C* and 2, *C*), with significant pain reduction; however, a new erosion, not treated with timolol, appeared on the right breast (Fig 1, *C*).

**Fig 1:**
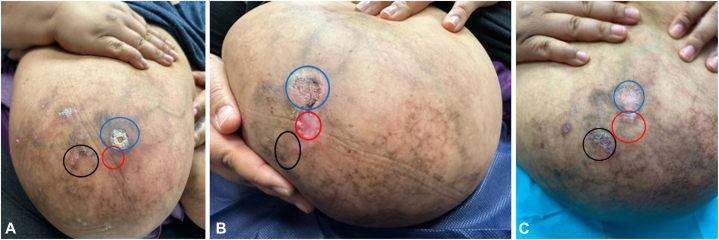
Clinical progression of right breast lesions. **A,** Right breast with livedoid skin changes and crusted erosions at initial visit—the *blue circle* represents the initial erosion, the *red circle* represents the punch biopsy site, and the *black circle* represents an adjacent area that was initially less concerning. **B,** Right breast erosions with partial re-epithelialization after starting oral pentoxifylline 400 mg once daily for approximately 1 month, as well as the biopsy site (*red circle*). **C,** Right breast with healed erosions (re-epithelialization) after adding topical timolol 0.5% solution twice daily to the regions represented by the *blue* and *red circles*, in addition to pentoxifylline 400 mg once daily for 6 weeks; the region represented by the *black circle* was not treated with topical timolol, which developed into a new crusted erosion.

**Fig 2:**
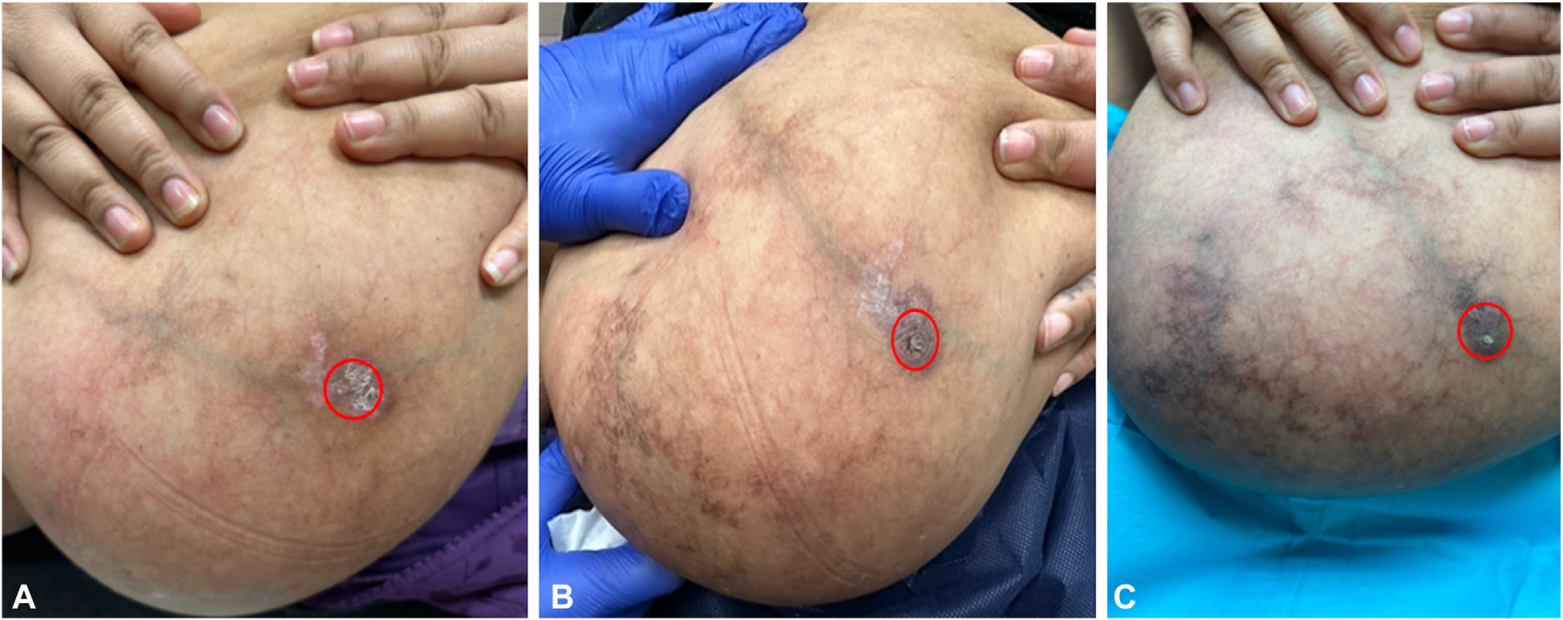
Clinical progression of left breast lesions. **A,** Left breast with livedoid skin changes and crusted erosion, represented by the *red circle*, at initial visit. **B,** Left breast erosion (*red circle*) with partial re-epithelialization after starting oral pentoxifylline 400 mg once daily for approximately 1 month. **C,** Left breast with healed erosion, increased fibrotic tissue, and improved skin integrity after adding topical timolol 0.5% solution twice daily to the region represented by the *red circle*, in addition to pentoxifylline 400 mg once daily, for 6 weeks.

**Fig 3:**
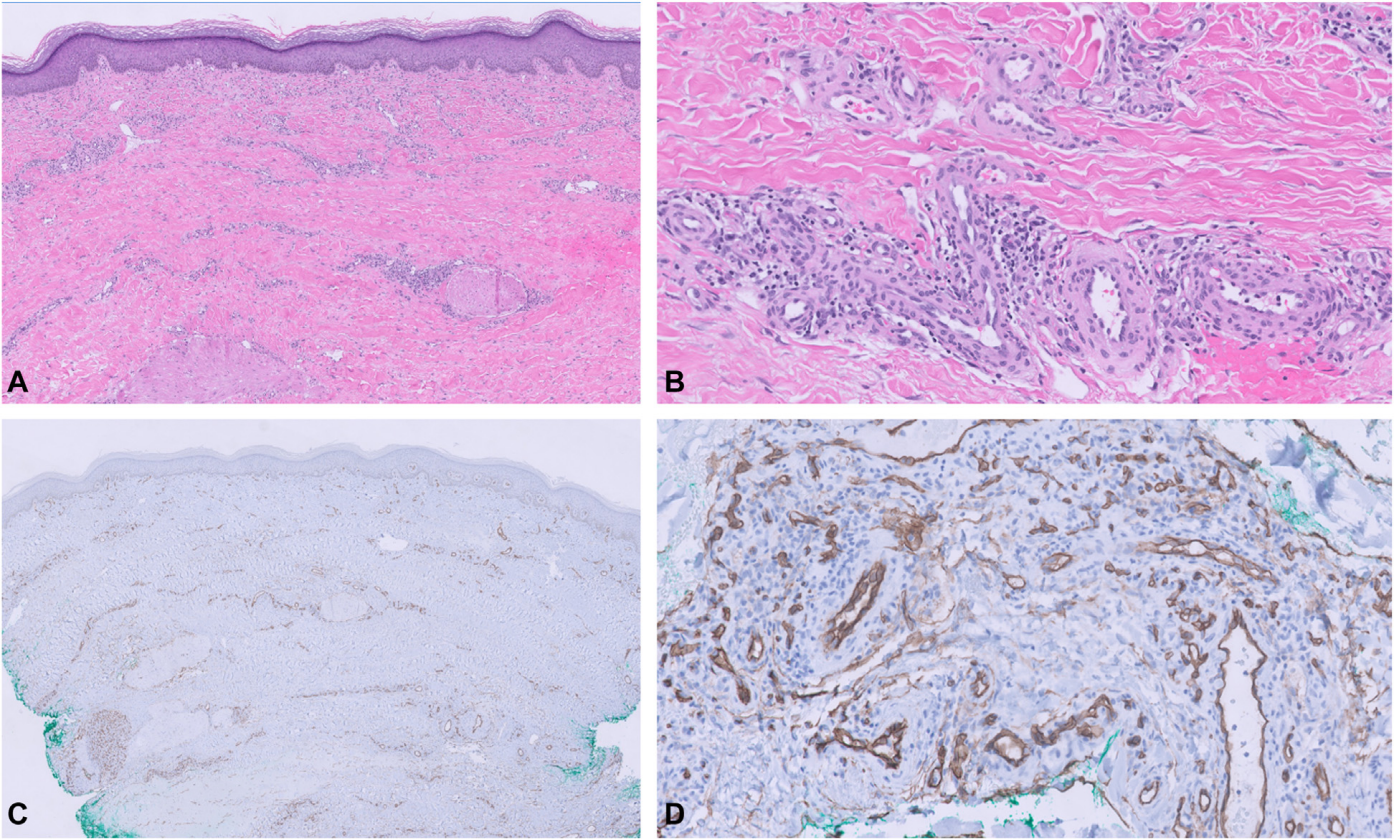
Histopathology of diffuse dermal angiomatosis of the right breast. **A,** Punch biopsy of the right breast showing diffuse proliferation of vessels involving the full thickness of the dermis, extending to the subcutaneous tissue (Hematoxylin and eosin stain; original magnification: 2×). **B,** High-power view shows that the vessels are lined by bland spindle-shaped endothelial cells (Hematoxylin and eosin stain; original magnification: 20×). **C,** CD34 immunostain highlights the diffuse proliferation of vessels involving the full thickness of the dermis (Immunohistochemistry; original magnification: 2×). **D,** High-power view shows the positive CD34 immunostain within the endothelial cells (Immunohistochemistry; original magnification: 20×).

COVID-19 has been linked to hypercoagulability and thrombotic complications. Up to 20.4% of patients may experience cutaneous involvement, including livedo, purpura, and necrosis.^4^ COVID-19 histologic features reveal perivascular inflammatory infiltrate with small-vessel thromboses.^4^ Conversely, a diffuse proliferation of bland endothelial cells throughout the dermis is characteristic of DDA-B.^2^ COVID-19 likely worsened the subclinical DDA-B in our high-risk patient with macromastia and obesity.

DDA-B management includes lifestyle changes, anticoagulants, and topical steroids. Breast reduction is considered the definitive solution.^3^ However, left untreated DDA-B has not been shown to spontaneously regress.^3^ Although pentoxifylline has been used for DDA-B, its efficacy appears limited.^3^ Thus, topical β-blockers were considered, given their vasoconstrictive, antivasoproliferative properties and their success in treating hemangiomas, pyogenic granuloma, and Kaposi sarcoma.^5^

This case of COVID-19-associated DDA-B expands on the inventory of COVID–19-associated dermatoses. Patients with large breasts and prior COVID-19 infection presenting with livedo and ulcers should be evaluated for DDA-B. Additionally, to our knowledge, we present the first case of DDA-B that demonstrated remarkable clinical improvement, including pain reduction, to the combination of pentoxifylline and topical timolol. Developing a new erosion at an untreated site supports that topical timolol and pentoxifylline may promote the healing of pre-existing erosions but is not a cure.

## Conflicts of interest

None disclosed.
